# Supplementary material for: Universal Health Coverage and Preparedness Payoffs: Global COVID-19 Vaccination Rates
Source: Vaccines (Basel). 2025 Apr 23;13(5):443. doi: 10.3390/vaccines13050443 (PMC12115366; doi:10.3390/vaccines13050443)
Supplement: Supplementary file 1 [file vaccines-13-00443-s001.zip › Supplementary_UHC and preparedness.pdf]

Supplementary S1

Table S1. Variable Definitions and Sources.

Variable	Source	Definition
Vaccination Rate	Our World in Data[1]	Percent of the population who received all doses prescribed by the initial vaccination protocol in each country.
State Parties Self-Assessment Annual Reporting (SPAR)	WHO[2]	Average score of the SPAR's13 capacities
Universal Health Coverage (UHC) index	WHO [3]	Average scores of the UHC's four sub-indices
Population above 65	WHO [4]	Percent of population above age 65
Country income classification	WHO [4]	Divided by four income groups – low, lower-middle, upper-middle, and high.

[1] Mathieu E, Ritchie H, Ortiz-Ospina E, Roser M, Hasell J, Appel C, et al. A global database of COVID-19 vaccinations. Nat Hum Behav. 2021 Jul 1;5(7):947–53.
[2] World Health Organization. States Party self-assessment annual reporting tool second edition [Internet]. 2022 [cited 2024 Dec 18]. Available from: https://extranet.who.int/e-spar/
[3] World Health Organization, The World Bank. Tracking universal health coverage: 2021 global monitoring report. Geneva: World Health Organization; 2021. Available from: https://www.who.int/publications/i/item/9789240040618
[4] World Health Organization. Global Health Expenditure Database [Internet]. 2024 [cited 2024 Dec 18]. Available from: https://apps.who.int/nha/database

Supplementary S2: Analytical Procedures

Data Management

Data from different sources were combined into a single spreadsheet, with countries matched by name and code. In cases of missing data, analyses were conducted using only the countries with available data for all variables of interest. The sample size is noted for each analysis to account for these variations. No data transformations were performed on the variables before analysis.

Statistical Analysis

All statistical analyses were performed using R version 4.3.2 in the RStudio environment. Our analytical approach incorporated three main components: descriptive statistics, correlation analyses, and multiple linear regression models. We calculated means and interquartile ranges for descriptive statistics for all variables and examined trends in vaccination rates across different country income groups over time.

The correlation analysis assessed bivariate relationships between preparedness indicators (SPAR and UHC indexes) and vaccination rates at different time points. In subsequent analyses, we also tested correlations between independent variables to understand their relationships and potential interactions.

Multiple Linear Regression Models

Model development followed a systematic approach based on hypothesized relationships between COVID-19 vaccination rates and predictor variables. Variables were selected to include preparedness indexes, population demographics (age), and country income classification. Multicollinearity was assessed in all regression models. We included an interaction term (UHC × SPAR) in relevant models to address potential multicollinearity between SPAR and UHC indexes. In our final selection process, we prioritized models with fewer predictor variables and higher adjusted R-squared values.

The general form of our main regression model was:

$$Vaccination\ Rate = \beta 0 + \beta 1(UHC) + \beta 2(SPAR) + \beta 3(UHC \times SPAR) + \beta 4(Age65+) + \beta 5(IncomeGroup) + \varepsilon$$

In this model, Vaccination Rate represents the percentage of population fully vaccinated, UHC is the Universal Health Coverage index, SPAR is the State Parties Self-Assessment Annual Reporting index, Age65+ represents the percentage of population aged 65 and above, and Income Group is a categorical variable for country income classification. We conducted separate analyses for vaccination rates in October 2021 and August 2022 to examine how relationships between these variables evolved over time.

Supplementary S3: the relationship between SPAR index and COVID-19 vaccination rates

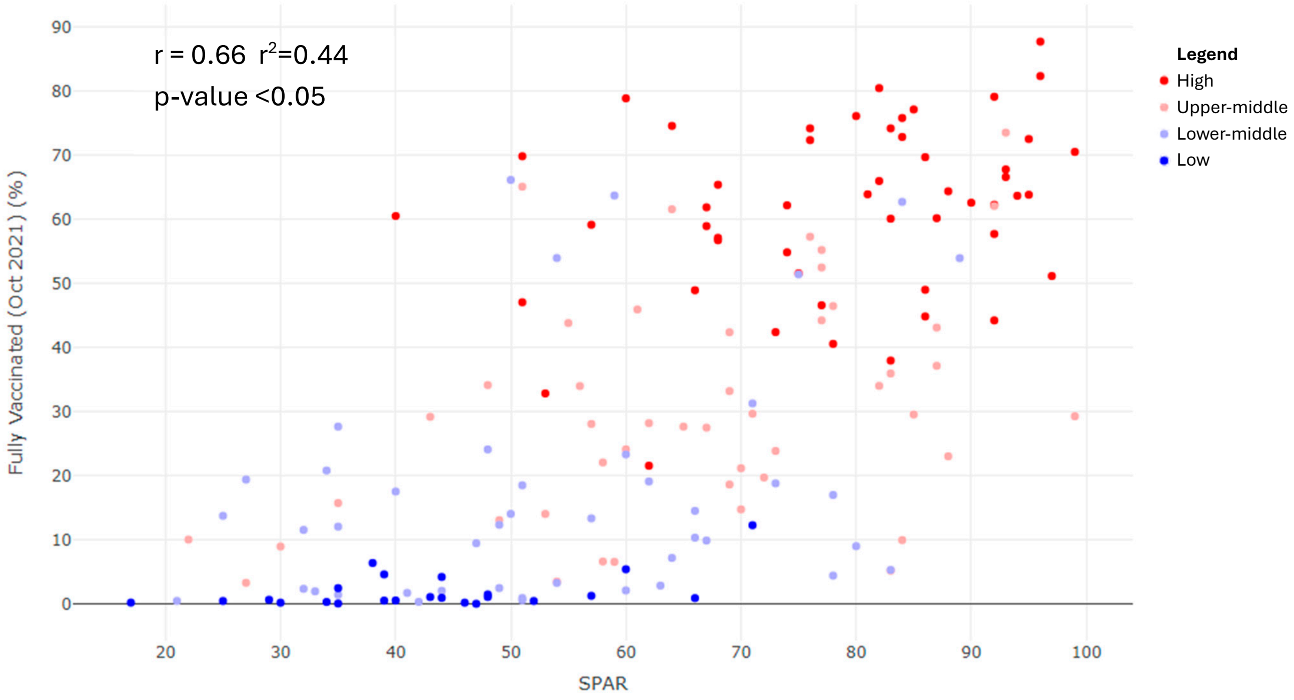

Figure S1. the relationship in October 2021 (n = 172).

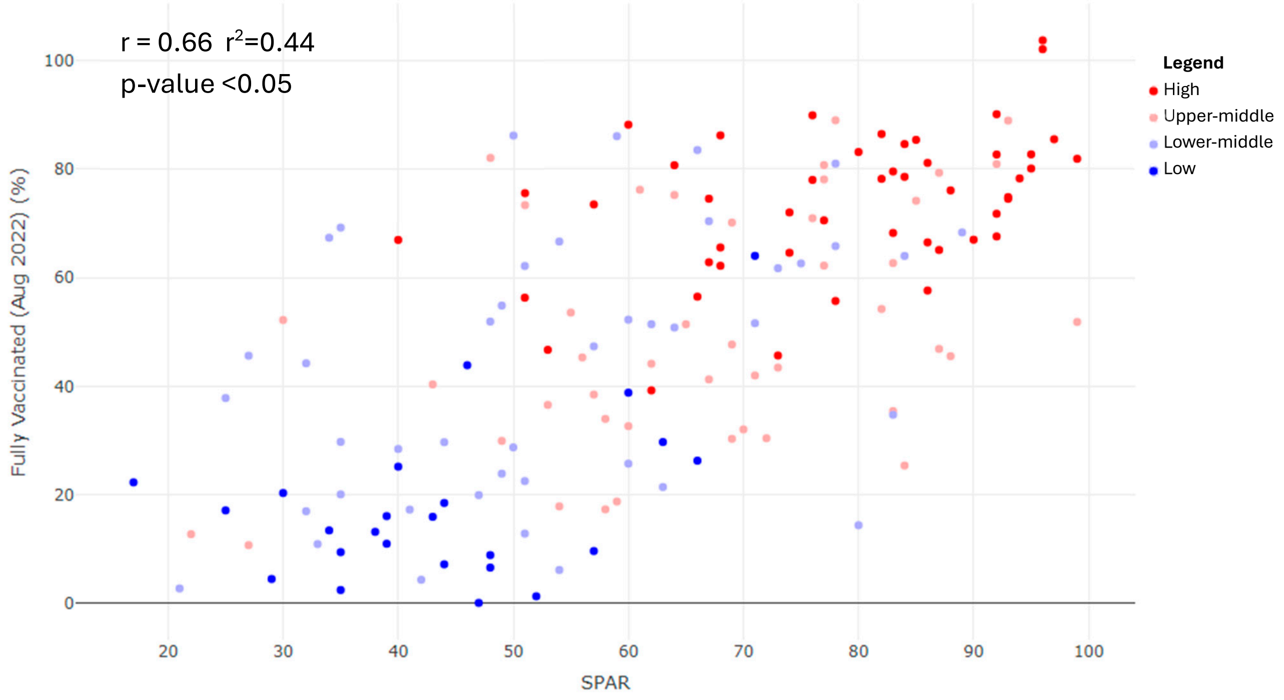

Figure S2. the relationship in August 2022 (n = 167).

Supplementary S4: regression results, including the interaction between UHC & SPAR

Table S2. Multiple Linear Regression Results for Predicting COVID-19 Vaccination Rates by October 2021 (n=162).

	**Model 1**	**Model 2**	**Model 3**
**Predictor**	**B (95% CI)**	**B (95% CI)**	**B (95% CI)**
**UHC**	0.81‡ (0.56 to 1.06)		0.75‡ (0.26 to 1.25)
**SPAR**		0.34‡ (0.19 to 0.49)	
**UHC X SPAR**			0.12 (-0.25 to 0.49)
**Above 65**	-0.28 (-0.74 to 0.19)	0.12 (-0.37 to 0.60)	-0.26 (-0.74 to 0.22)
**Income group (ref: Low)**			
**Lower-Middle**	3.22 (-4.50 to 10.94)	11.34† (3.67 to 19.01)	2.96 (-5.14 to 11.06)
**Upper-Middle**	8.69 (-0. 51 to 17.89)	19.66‡ (11.13 to 28.12)	6.42 (-3.47 to 16.32)
**High**	31.53‡ (20.39 to 42.67)	45.70‡ (35.23 to 56.18)	29.59‡ (17.47 to 41.71)
**Intercept**	-31.22	-12.92	-34.00
**Adjusted R-Squared**	0.71	0.69	0.72

Note. B = Unstandardized Coefficient, (95% CI) = 95% Confidence Interval.

Significance levels: '*' < 0.05; '†' < 0.01; '‡' < 0.001.

Table S3. Multiple Linear Regression Results for Predicting COVID-19 Vaccination Rates by <u>August 2022</u> (n=162).

	**Model 1**	**Model 2**	**Model 3**
**Predictor**	**B (95% CI)**	**B (95% CI)**	**B (95% CI)**
**UHC**	1.19‡ (0.87 to 1.51)		0.93‡ (0.35 to 1.52)
**SPAR**		0.53‡ (0.35 to 0.71)	
**UHC X SPAR**			0.33 (-0.11 to 0.77)
**Above 65**	-0.61 (-1.25 to 0.03)	0.07 (-0.59 to 0.73)	-0.45 (-1.10 to 0.20)
**Income group (ref: Low)**			
**Lower-Middle**	9.02 (-0.39 to 18.44)	20.06‡ (10.95 to 29.16)	8.44 (-1.09 to 17.97)
**Upper-Middle**	5.80 (-5.57 to 17.18)	19.66‡ (9.36 to 29.96)	1.40 (-10.43 to 13.23)
**High**	18.87† (4.96 to 32.79)	36.23‡ (23.27 to 49.19)	14.08 (-0.62 to 28.78)
**Intercept**	-29.94	-5.71	-33.79
**Adjusted R-Squared**	0.58	0.56	0.63

Note. B = Unstandardized Coefficient, (95% CI) = 95% Confidence Interval.

Significance levels: '*' < 0.05; '†' < 0.01; '‡' < 0.001.
